# Adapted Acoustic CR Neuromodulation in Patients With Chronic Tonal Tinnitus and Hearing Loss

**DOI:** 10.3389/fmed.2018.00288

**Published:** 2018-10-10

**Authors:** Hannes Wurzer, Christian Hauptmann

**Affiliations:** ^1^Tinnitus Center, Munich, Germany; ^2^Desyncra Operating GmbH, Bad Neuenahr-Ahrweiler, Germany

**Keywords:** tinnitus, neuromodulation, acoustic stimulation, therapy, chronic disease

## Abstract

Chronic tonal tinnitus is often accompanied by sensorineural hearing loss which is associated with altered tuning curves and bandwidth of alternating masking. In this feasibility study the so-called hearing threshold adapted coordinated reset (HTA-CR) neuromodulation was investigated. This method is based on CR neuromodulation, which has been demonstrated to be an effective treatment for chronic tonal tinnitus. It applies four stimulation tones that are determined by the patient's individual tinnitus frequency and hearing impairment. The HTA-CR neuromodulation was programmed to the Desyncra™ for Tinnitus Therapy System and treatment was applied to 25 patients for 4 months on average and 4 h daily. Regular check-ups were done every 4–6 weeks. Therapy outcome was assessed by the tinnitus questionnaire (*Tinnitusfragebogen*, TF) as per Goebel and Hiller. After 4 months the mean TF score was reduced by 27.4%. A reduction of ≥ 15 points was found in 40% of the patients while for further 32% of the patients a reduction of 6–14 points was found. Thus, a positive response rate of 72% was observed after 4 months of HTA-CR neuromodulation. Our results suggest that HTA-CR neuromodulation might be at least comparable to standard CR neuromodulation providing another effective therapeutic option for the treatment of chronic tonal tinnitus.

## Introduction

Chronic tinnitus is an otorhinolaryngological disease affecting ~10–15% of the general population in industrialized countries (1). The permanent perception of sound in the absence of a corresponding sound source is often associated with hearing loss secondary, among others, to noise exposure or aging. Recent studies using advanced imaging techniques and functional approaches indicated that chronic tinnitus is a neural consequence of acoustic acquired sensory deprivation (2–5) leading to an imbalance of excitatory and inhibitory neural networks in the central auditory pathway. These alterations result in elevated spontaneous activity (6) and synchronization of neurons (7, 8) in the auditory cortex, virtually a “tinnitus generator,” which is perceived as tinnitus. An overview of involved neuropathophysiological circuits has been published recently by Rauschecker et al. (9).

Because only 20% of patients with chronic tinnitus are affected by a severe impairment of quality of life (10) it is assumed that the limbic system, the “emotional brain,” and the vegetative neuronal network including the *formatio recticularis* impact the level of suffering experienced by patients (11–14). This aspect is recognized and implemented in various therapeutical approaches for tinnitus treatment such as use of psychotropic drugs, relaxation techniques (e.g., progressive relaxation, Tai Chi, etc.), physiotherapy, cognitive behavioral therapy (CBT), and tinnitus retraining therapy (TRT) (15–17). In particular in Germany, TRT has been modified substantially and is often combined with a sound therapy or tinnitus noisers, i.e., noise generators used to mask or cover up the tinnitus sensation (18). So far, only for CBT efficiency in the treatment of tinnitus has been demonstrated in a randomized clinical trial (19).

Various therapies for chronic tinnitus have been developed aiming to manipulate the hyperactive “tinnitus generator” in the auditory cortex by neuromodulation via acoustic stimulation. Beside some special German methods like the Heidelberger music therapy (20) and the tinnitus-centered music therapy (TIM) developed by Cramer (21), tinnitus noisers and maskers are widely used (18). However, the recommendations for therapy of chronic tinnitus with these instruments differ substantially, and sufficiently large randomized clinical trials to demonstrate the efficacy of commonly used interventions are lacking (22). Moreover, none of the in Germany currently available tinnitus noisers is able to efficiently mask tinnitus tones with frequencies above 11 kHz. Thus, more advanced noisers have been developed which offer additional acoustic stimulation such as sounds of rainfall, ocean waves or wind chime.

Another approach of acoustic neuromodulation is the so-called tailor-made notch music therapy (TMNMT), where patients listen to music from which a frequency band of one octave around their individual tinnitus frequency had been removed (23). In this way neurons in the auditory cortex coding the tinnitus frequency are subject to lateral inhibition by neighboring neurons, whereas the afferent input to these neurons is negligible. Results from magnetoencephalography (MEG) have shown that the auditory evoked cortex activity was reduced after 12 months and patients reported reduced subjective tinnitus loudness (23). However, a recent double-blind randomized controlled trial applying TMNMT for 3 months on 100 patients with chronic tonal tinnitus failed to show an improvement on tinnitus distress assessed by the Tinnitus Questionnaire (TQ) (24).

A different approach is the acoustic coordinated reset (CR) neuromodulation, which was developed by the Research Center Juelich, Germany, and is based on theoretical and clinical studies (25–28). The method aims to desynchronize the synchronous hyperactive neuron population coding the tinnitus frequency by sequential stimulation of different subpopulations of the target population (25, 26, 29). For this purpose, an extensive pitch-matching procedure is used to determine the individual tinnitus frequency (30). Four acoustic stimulation tones are then generated based on a computationally developed CR algorithm. These tones are of different frequencies centered around the patient's tinnitus frequency, and their loudness is individually adapted to the loudness of the tinnitus. Via dedicated sound generators and ear phones the patients are exposed to the acoustic stimulation for several hours per day. The first blinded study reported by Tass et al. in 2012 with 63 patients proved the safety and showed clinical efficacy of CR neuromodulation (28). Since then several clinical studies with more than 500 patients suffering from chronic tonal tinnitus have been conducted. The results revealed that 60–75% of patients treated with CR neuromodulation respond well to this therapy as evident from decrease in tinnitus questionnaire scores and improvement of visual analog scale (VAS) scores for tinnitus loudness and annoyance by ~40% (14, 31, 32, Wurzer et al. submitted). Importantly, these improvements are persistent and stable. MEG and electroencephalography (EEG) data revealed specific alterations in patients with chronic tonal tinnitus such as increased oscillatory power in the delta frequency range and decreased alpha power in the auditory cortex region (12, 33, 34). Upon CR neuromodulation a normalization of the EEG pattern was observed in therapy responding patients (14, 35). Despite these promising results for the majority of chronic tinnitus patients ~30% of these patients experience no improvement of their tinnitus after CR neuromodulation, the so-called non-responders. Accordingly, efforts are made to further optimize the therapy.

The currently used CR neuromodulation stimulation tones depend exclusively on the tinnitus frequency. Theoretically, a 25–30% overlap of the stimulation tone's tuning curves would be optimal. However, several years of clinical experience support the notion that the patient's hearing ability should be considered in the individual adaption of the CR neuromodulation therapy. Sensorineural hearing loss is known to be associated with altered tuning curves, altered bandwidth of alternating masking and a changed discrimination that might be relevant for the acoustic overlap of the stimulation tones (36). Therefore, in this feasibility study for the first time the patient's tinnitus frequency, the intensity of the adjusted stimulation tones as well as his/her individual audiogram were taken into account when calculating the stimulation tones for the so-called hearing threshold adapted CR neuromodulation therapy (HTA-CR). Basically, the intensity of the stimulation tones was identified to be responsible for the neuronal recruitment. Stronger stimulation tones recruit more neurons and have therefore a flatter tuning curve (36). Since the individual subject is asked to adjust the stimulation such that the tones are audible the adjusted tone intensity strongly depends on the hearing threshold. The experiments done by Hopkins and Moore (36) provide the database linking the tone intensities and hearing loss with the shape of the tuning curve, which is used to calculate the adapted stimulation tones in an iterative approach. The simple study design lacks any control group, since the goal of this feasibility study was to test, if a hearing threshold adapted CR neuromodulation could be applied and to obtain first results which can be used as input for a larger, randomized and controlled study.

The Desyncra™ for Tinnitus medical devices (30) used in this study were programmed accordingly. The primary objective of this study was to verify a clinically significant improvement of chronic tinnitus by this adapted CR neuromodulation within 3–4 months, which was assessed by the tinnitus questionnaire (*Tinnitus-Fragebogen*, TF) as per Goebel and Hiller [German version of Hallam's TQ (37)].

## Materials and methods

### Study participants

Twenty-five patients attending a specialized consultation session for tinnitus were enrolled in this clinical study on acoustic CR neuromodulation, which was conducted in a specialized center run by an ear, nose and throat (ENT) specialist located in Munich, Germany. All patients were comprehensively informed about the scope, aim, benefits, and risks of study participation, and a written informed consent was obtained from all participants according to the Declaration of Helsinki and Good Clinical Practice. The study was approved by the relevant ethics committee (Ethics Committee of the Bavarian State Medical Association, BLAEK 2016-136). The inclusion and exclusion criteria are listed in Table 1.

**Table 1:** Overview of inclusion and exclusion criteria.

**Inclusion criteria**
1. Age ≥ 18 years 2. Primary chronic tinnitus ≥3 months [defined by AAO HNSF guidelines (40)] 3. Tonal tinnitus 4. Tinnitus frequency of 0.4–10 kHz (in exceptional cases up to 12 kHz) 5. TF score > 30 (i.e., at least severity grade II) 6. Able to hear all stimulation tones 7. Commitment to wear the device for 4–6 h/day 8. No other tinnitus treatment in the period of the clinical investigation
**Exclusion criteria**
1. Secondary / somatic tinnitus [defined by AAO HNSF guidelines (40)] 2. Atonal, pulsatile, or intermittent tinnitus 3. Hearing loss > 70 dB HL between 0.25 to 10 kHz 4. Tinnitus main frequency differs >10% between right and left ear 5. Health related or other reasons that might prevent the patient to complete the study 6. Use of medication that might cause tinnitus, i.e., daily high-dosed NSAIDs (≥1,000 mg/d) and salicylates at doses higher than for cardio-protection, loop diuretics, chemotherapy agents (e.g., cisplatin) 7. Permanent conductive hearing loss ≥15 dB for more than two frequencies in one ear 8. Persistent eardrum defect 9. Atresia or malformation of the outer ear 10. Acute otorrhoea 11. Acute or fast progressing hearing loss within the last 90 days 12. Severe psychiatric disorders

Screening data obtained during the initial examination (visit 0) are summarized in Table 2. The patients' age was between 21 years and 82 years (mean 51.3 years); the male to female proportion was 16–9.The hearing ability ranged from normal to severe hearing loss. The average hearing loss was calculated from the sum of hearing loss at 0.25, 0.5, 1, 2, 4, 6, and 8 kHz. The frequencies 6 and 8 kHz were included in the calculation taking the hearing loss in the high frequency range of the patients into account. Patients were classified into three hearing groups: group 1 with normal hearing ability (mean hearing impairment < 20 dB); group 2 with mild hearing loss (mean hearing impairment between 20 and 40 dB), and group 3 with moderate to severe hearing loss (mean hearing impairment > 40 dB, Table 2) (31, 38). Five patients using hearing aids were included in the study. Chronic tinnitus duration was between 1 and 35 years (median 8.0 years). The mean TF score at inclusion was 50.4 ± 12.9 points, and most of the patients suffered from tinnitus with severity grade ≥ 3. Moreover, all patients have been treated before study start with at least two different therapeutic approaches (cortisone infusion, noisers, acupuncture, etc.) without success.

**Table 2:** Screening data of study population (*n* = 25).

**Variable**	**Mean ±SD (range)**
**AGE (YEARS)**	
Overall	51.3 ± 13.6 (21–82)
**By Hearing Impairment**
Group 1 (< 20 dB, *n* = 11)	46.3 ± 12.2 (21–64)
Group 2 (20–40 dB, *n* = 7)	47.9.4 ± 6.8 (39–59)
Group 3 (> 40 dB, *n* = 7)	62.6 ± 15.5 (37–82)
Tinnitus duration (years)	8.0^*^ (1–35)
TF score	50.4 ± 12.9 (32–73)
	**Number of patients (%)**
**SEX**	
# Male	16 (64.0%)
# Female	9 (36.0%)
**TINNITUS SEVERITY**	
Grade 1 (0–30 points)	0 (0.0%)
Grade 2 (31–46 points)	12 (48.0%)
Grade 3 (47–59 points)	6 (24.0%)
Grade 4 (60–84 points)	7 (28.0%)

* *Value given as median*.

### Description of the medical device

**Figure 1:**
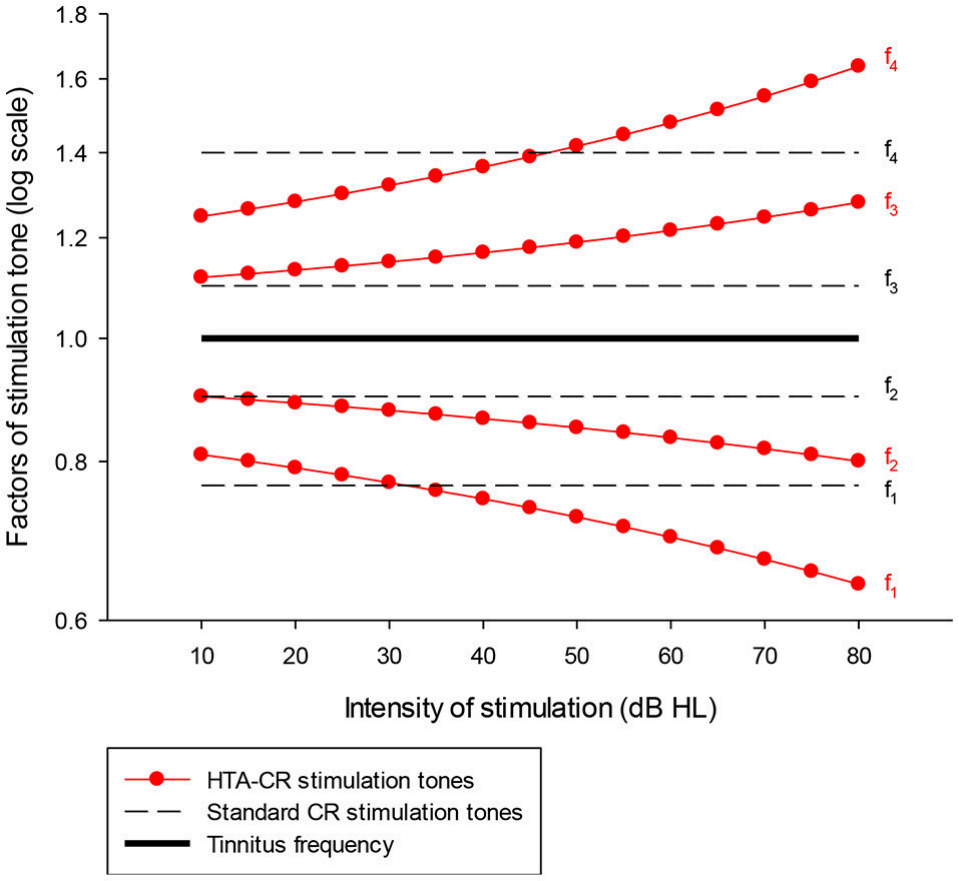
Graphic presentation of the factors used to determine the frequency of the four stimulation tones (f_1_-f_4_) for HTA-CR neuromodulation (line with dots) and standard CR neuromodulation (dash line). It is assumed that the stimulation tones are perceived ~10 dB above the individual hearing threshold. A patient with normal hearing ability hears the tones with 10 dB HL whereas a patient with a hearing loss of 40 dB at the tinnitus frequency hears the tones with 50 dB HL. Taking the last case as example, the factors to determine the stimulation tones are 0.72 (tone 1 = f_1_), 0.85 (f_2_), 1.19 (f_3_), and 1.42 (f_4_) using the HTA-CR neuromodulation corresponding to a deviation of −5.58% (f_1_), −5.51% (f_2_), 8.20% (f_3_), and 1.18% (f_4_) compared to the standard CR neuromodulation factors. These factors are then multiplied with the individual tinnitus frequency to calculate the frequency of the stimulation tones.

For HTA-CR neuromodulation the Desyncra™ for Tinnitus Therapy System was used, which is a Class IIa medical device (CE-0123, certified 2016 by TÜV Süd, Germany; FDA certified 2016 by FDA, no. K151558). To adapt the system for HTA-CR neuromodulation, the relation of the stimulation tones to each other and to the individual tinnitus frequency was modified taking into account the individually adjusted tone intensity and measured hearing loss (Figure 1). The algorithm of time and tone sequence as used for the standard acoustic CR neuromodulation was left unaltered. The adjustments were done according to the guidelines and controlled after 4–6 weeks. The patients were asked to use the device daily for 4 h on average. The total duration of the study was 4 months.

### Study conduct

During the initial examination (visit 0) medical and tinnitus history was assessed, a medical examination of ear, nose, throat, and the stomatognathic system was performed as well as manual examination of the cervical spine. A hearing threshold and high frequency (11–18 kHz) audiogram was recorded, and a speech audiogram and evaluation of hearing aid performance was performed if required. Moreover, measurement of impedance and otoacoustic emissions, repeated tinnitus pitch matching and tuning curve measurement at 1 kHz were conducted. Patients completed the TF and, if found eligible and interested in study participation, they received the EC approved patient information and informed consent form.

At the start of the study (= visit 1) patients were fully informed about the study and signed the informed consent form. ENT examinations, audiogram recording, and tinnitus pitch matching procedures were repeated, and patients completed the following questionnaires to assess various aspects of the tinnitus: TF, *Tinnitus-Beeintraechtigungsbogen* (TBF-12), Tinnitus Handicap Inventory (THI, German version), and Tinnitus Functional Index (TFI, only available in English). The TFI contains numeric rating scales (NRS) which were used to assess tinnitus loudness (NRS-L) and annoyance (NRS-A). The baseline scores of the questionnaires are summarized in Table 3. After completing all study assessments of visit 1 patients received their individually programmed mobile device and were also provided with detailed instructions and information relating to device usage at home.

**Table 3:** Baseline scores of questionnaires (*n* = 25). NRS-L and NRS-A scores were taken from the TFI questionnaire.

**Variable**	**Mean ±SD (range)**
TF score	44.9 ± 12.1 (27–71)
TBF-12 score	13.6 ± 4.7 (5–23)
THI score	52.5 ± 20.9 (10–92)
TFI score	55.1 ± 18.7 (11.2–86.6)
NRS-L score	6.5 ± 2.2 (1–10)
NRS-A score	5.4 ± 2.7 (1–9.5)

Check-ups were performed after 5 ± 1 weeks (= visit 2) and 9 ± 1 weeks (= visit 3). During the visits questionnaires were repeated, tinnitus pitch matching performed and the stimulation tones adjusted to the latest tinnitus frequency. Adverse events, adverse device effects and general problems with the device were collected.

The final visit took place after 16 ± 2 weeks (visit 4) with determination of the latest tinnitus frequency, tuning curve assessment, audiogram control, and completion of TF, TBF12, THI, and TFI by the patient.

### Outcome variables

For all questionnaires the scores were calculated according to the instructions coming with the questionnaire.

To evaluate the therapy outcome TF score change from visit 1 to visit 4 was calculated (primary endpoint). An at least 15-point reduction in TF score during the study period (16 ± 2 weeks) was regarded as success (“Winner,” i.e., great responder), a reduction of 6–14 points was defined as an improvement (“Responder”). If a change between −5 and +5 points was observed, the result was classified as unchanged (“Non-responder”), and increases by at least 6 points were judged as deterioration (“Loser,” i.e., worsened case).

### Statistical analysis

Scores obtained from questionnaires are summarized as mean ± standard deviation (SD). Changes from baseline (scores before treatment minus scores after treatment) concerning scores for all questionnaires were analyzed by paired Student's *t*-test. To assess potential relationships between continuous variables (age, tinnitus duration, tinnitus severity, and therapy outcome) Pearson's coefficient of correlation (r) was calculated. Response rates were compared between sex groups and between groups of hearing impairment by use of Fisher's exact test for contingency tables. All statistical analyses were performed by M.A.R.C.O. GmbH & Co.KG, Institute for Clinical Research and Statistics (Düsseldorf, Germany) using SAS® version 9.3. A *p* < 0.05 was considered statistically significant.

## Results

All patients completed the study. Due to technical reasons (i.e., vacation dates) the duration of the study was slightly longer than originally planned. On average patients used the Desyncra™ for Tinnitus Therapy System for 16 weeks (from visit 1 to visit 4).

At baseline a positive correlation was found between age and duration of the tinnitus (r = 0.43, *p* = 0.033), whereas age and tinnitus severity were not correlated (r = 0.31, *p* = 0.13).

**Figure 2:**
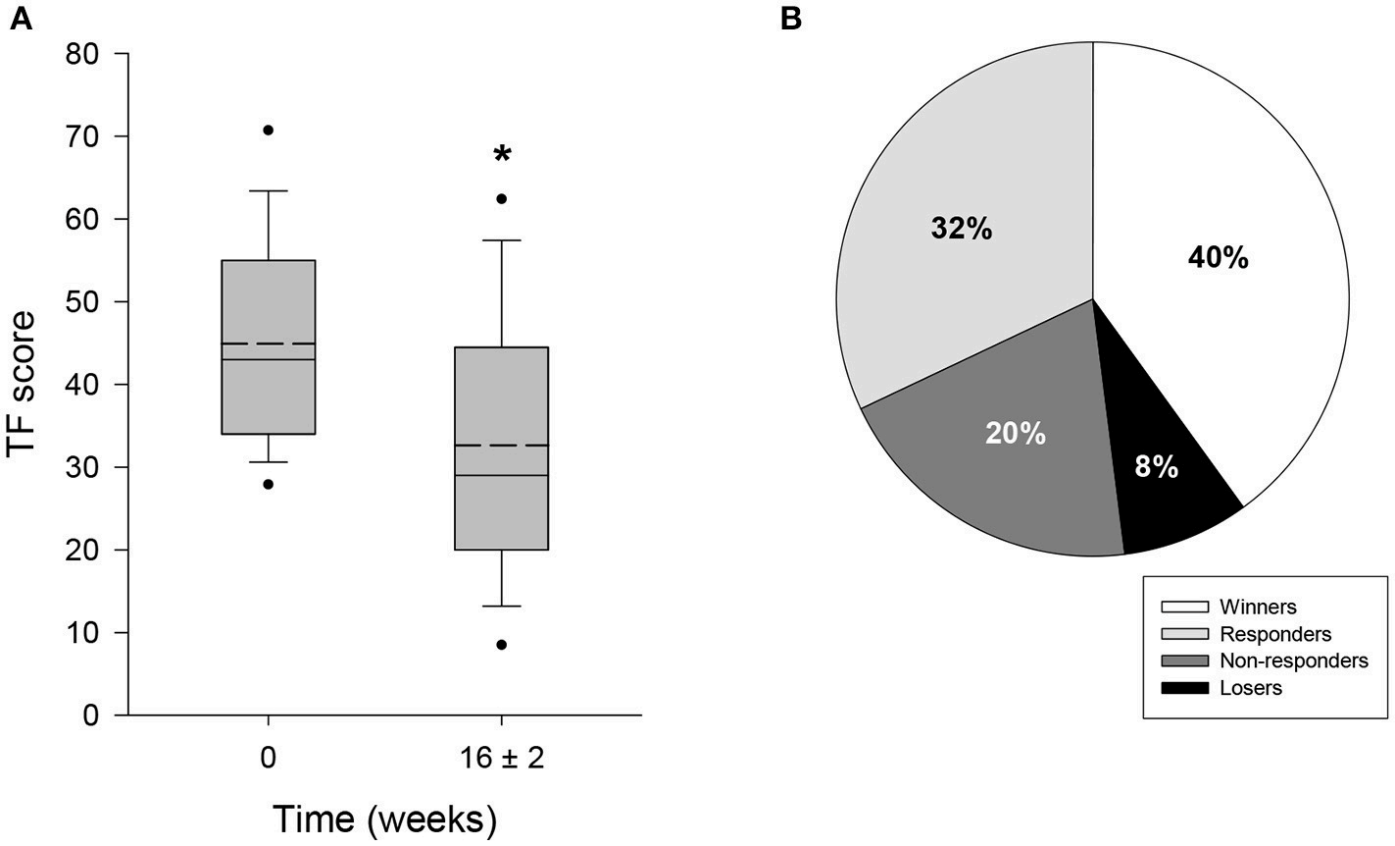
Evaluation of tinnitus by TF questionnaire. 25 patients participated in the study and completed TF at visit 1 (baseline, 0 weeks) and visit 4, i.e., after 16 ± 2 weeks of therapy with HTA-CR neuromodulation. **(A)** Box plots of TF scores showing median (solid line), mean (dash line), 5th and 95th percentile; *n* = 25, ^*^p < 0.001 (paired *t*-test). **(B)** Percentage of patients classified as “Winners” (TF score reduced by ≥15 points), “Responders” (TF score reduced by 6–14 points), “Non-responders” (change between −5 and +5 points), and “Losers” (TF score increased by ≥ 6 points).

HTA-CR neuromodulation resulted in a significant reduction of tinnitus and associated discomfort. At the beginning of the study, the mean TF score was 44.9 ± 12.1 (Table 3). At the end of the study it was reduced to 32.6 ± 15.5 points, corresponding to reduction by 27.4% (*p* < 0.001) compared to baseline (Figure 2A). As shown in Figure 2B, for 10 out of 25 study participants (40%) the TF score was strongly reduced by more than 15 points, which classified them as “Winners.” Moreover, for additional 8 out of 25 patients (32%) a reduction in the range of 6 to 14 points was observed (“Responders”). Thus, the overall response rate (“Winners” plus “Responders”) results to 72% in this study. No change of tinnitus was observed in 5 out of 25 patients (20% “Non-responder”) treated with HTA-CR neuromodulation while 2 patients (8%) experienced a deterioration of the pre-existing conditions (“Losers”).

Interestingly, mean TF scores were higher at the initial examination compared to study start (visit 0: 50.4 ± 12.9 points; visit 1: 44.9 ± 12.1 points; see Tables 2, 3), corresponding to a reduction of 10.9% (*p* = 0.002).

Table 4 shows descriptive statistics relating therapy outcome with baseline characteristics. Response to the therapy (i.e., improvement of TF score) did not seem to depend on age (r = 0.07, *p* = 0.733) and tinnitus duration (r = 0.31, *p* = 0.135), whereas it appears to increase with tinnitus severity (r = 0.36, *p* = 0.079). Response rates were not significantly different between male and female patients (62.5 vs. 88.9%, *p* = 0.355). With regard to the degree of hearing impairment, therapy response was not significantly different between the hearing groups (*p* = 0.65). Both in group 1 and group 3 more than 40% of the patients experienced such an improvement of TF score that they were classified as winners.

**Table 4:** Therapy outcome related to baseline characteristics.

	**Winners (*n* = 10)**	**Responders (*n* = 8)**	**Non-responders (*n* = 5)**	**Losers (*n* = 2)**
Mean age (range)	53.5 years (40–82)	47.8 years (21–71)	53.6 years (39–77)	48.5 years (37–60)
Median tinnitus duration (range)	8.5 years (1–35)	8.0 years (3–20)	7.0 years (3–18)	6.0 years (1–11)
**SEX**				
# male (*n* = 16)	7 (43.7%)	3 (18.8%)	4 (25.0%)	2 (12.5%)
# female (*n* = 9)	3 (33.3%)	5 (55.6%)	1 (11.1%)	0 (0.0%)
**TINNITUS SEVERITY (# PATIENTS)**				
Grade 1 (*n* = 2)	1 (50.0%)	1 (50.0%)	0 (0.0%)	0 (0.0%)
Grade 2 (*n* = 15)	5 (33.3%)	5 (33.3%)	3 (20.0%)	2 (13.3%)
Grade 3 (*n* = 6)	3 (50.0%)	1 (16.7%)	2 (33.3%)	0 (0.0%)
Grade 4 (*n* = 2)	1 (50.0%)	1 (50.0%)	0 (0.0%)	0 (0.0%)
HEARING IMPAIRMENT (# PATIENTS)^*^				
Group 1 (*n* = 11)	5 (45.5%)	4 (36.4%)	2 (18.2%)	0 (0.0%)
Group 2 (*n* = 7)	2 (28.6%)	3 (42.9%)	2 (28.6%)	0 (0.0%)
Group 3 (*n* = 7)	3 (42.9%)	1 (14.3%)	1 (14.3%)	2 (28.6%)

* *Refer to Table 2*.

**Figure 3:**
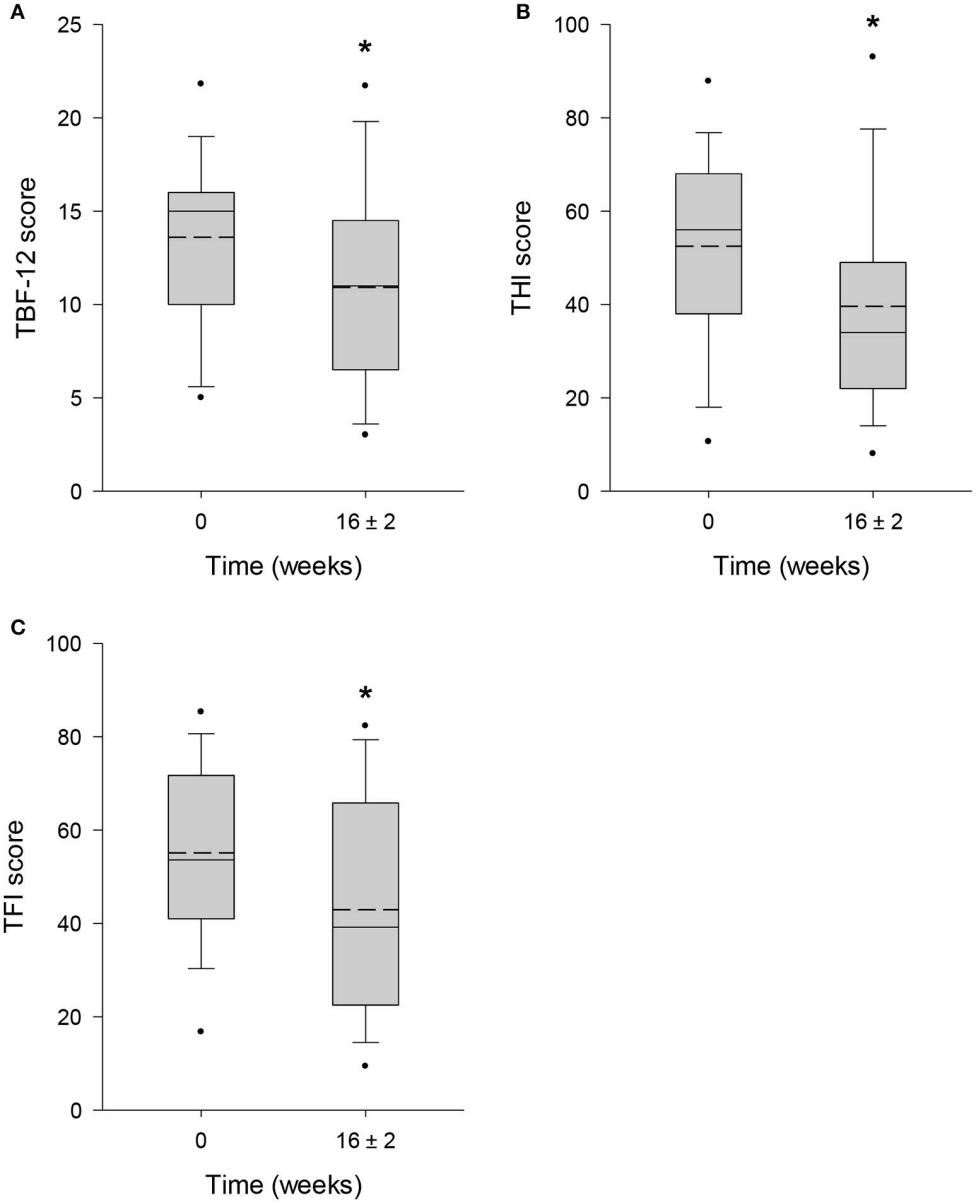
Evaluation of tinnitus by TBF-12 **(A)**, THI **(B)**, and TFI **(C)** questionnaires. All patients completed the questionnaires at visit 1 (baseline, 0 weeks) and visit 4 (16 ± 2 weeks). Results are shown as box plots with median (solid line), mean (dash line), 5th and 95th percentile; *n* = 25, ^*^p < 0.05 (paired *t*-test).

**Figure 4:**
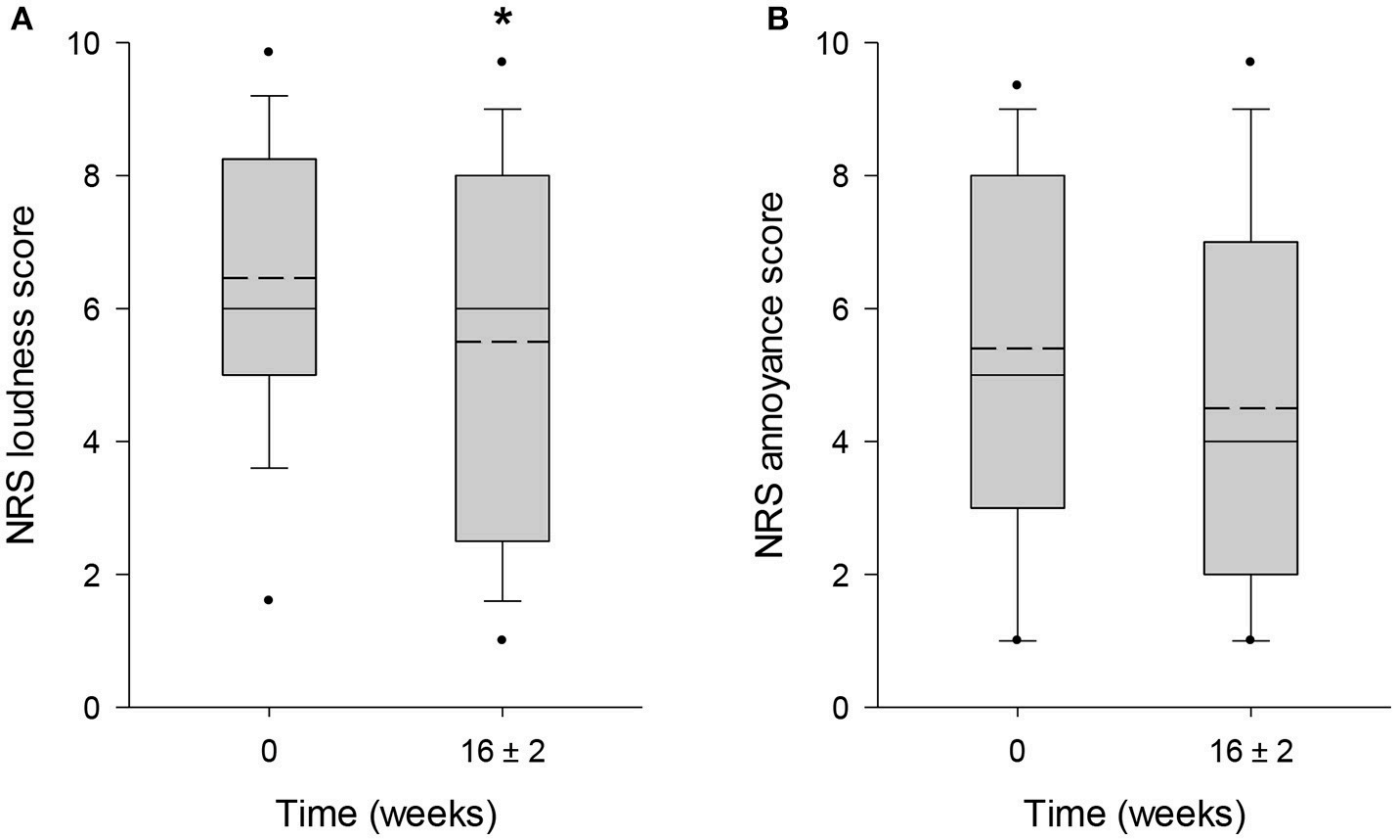
Evaluation of tinnitus by NRS for loudness **(A)** and annoyance **(B)**. Patients completed the scales as part of the TFI questionnaires at visit 1 (baseline, 0 weeks) and visit 4 (16 ± 2 weeks). Results are shown as box plots with median (solid line), mean (dash line), 5th and 95th percentile; *n* = 25; ^*^p < 0.05 (paired *t*-test).

The mean score obtained from TBF-12 decreased significantly by 19.7% (*p* = 0.006) from 13.6 ± 4.7 points at baseline to 10.9 ± 5.4 points after 16 ± 2 weeks of HTA-CR neuromodulation therapy (Figure 3A). For the THI questionnaire, with a maximum score of 100 points, a significant reduction of 24.5% (visit 1: 52.5 ± 20.9 points; visit 4: 39.6 ± 22.4 points; *p* = 0.013) was observed (Figure 3B). Similarly, a significant decrease by 22.1% (*p* = 0.003) from 55.1 ± 18.7 points to 42.9 ± 23.3 points was found for the TFI (Figure 3C). To assess tinnitus loudness and annoyance, NRS scores were evaluated. For NRS-L (Figure 4A), the baseline value at visit 1 was 6.5 ± 2.2 points and 5.5 ± 2.9 points at visit 4 corresponding to a decrease of 14.9% (*p* = 0.022). NRS-A scores were consistently reduced by 16.7% (visit 1: 5.4 ± 2.7 points; visit 4: 4.5 ± 2.8 points; *p* = 0.106) after 16 ± 2 weeks of HTA-CR neuromodulation therapy (Figure 4B).

During the study six adverse events defined as any untoward medical occurrence, and two serious adverse events (SAEs) were reported. Six out of 25 patients reported a temporary worsening of tinnitus symptoms, sensation of head pressure and headache. These side effects were reversible and rapidly disappeared after adjustment of the stimulation or daily stimulation duration. One patient experienced an acute psychosis (anxiety disorder) at the end of the study and was hospitalized for 14 days. Another patient had an unknown cyclothymia and experienced a serious depressive phase during the study resulting in hospitalization for several weeks. Both SAEs were evaluated as not related to the therapy with HTA-CR neuromodulation.

## Discussion

This study investigating HTA-CR neuromodulation with use of the Desyncra™ for Tinnitus therapy system is based on an adapted form of the standard CR neuromodulation, which was developed through many years of research at the Research Center Juelich, Germany. First results with standard CR neuromodulation from 2012 revealed response rates of 75% in patients with chronic tonal tinnitus (28), which has been confirmed in recent studies comprising meanwhile more than 500 patients (14, 31, 32, Wurzer et al. submitted). A larger double blind study to prove the effectiveness of this method has been carried out in England (RESET2, NCT01541969), but due to serious technical errors the results were not useable (39). Thus, new randomized double blind studies are currently being conducted. Until the results from these studies are available, the effectiveness of CR neuromodulation is critically evaluated in daily clinical practice using non-interventional study approaches (31, 32, Wurzer et al. submitted).

The stimulation tones used for standard CR neuromodulation are determined based on theoretical considerations and experimental results. In an outpatient setting, 67% of patients suffering from tonal tinnitus experienced significant tinnitus improvements after applying this method for 6–12 months (31, Wurzer et al. submitted). However, the altered processing of acoustic stimuli in the whole auditory system associated with sensorineural hearing loss and further experimental results suggest that the stimulation tones might and should be further optimized by adjusting them to the patient's individual tone thresholds. Thus, we applied this idea in our study using the individual stimulation tone intensity and the experimental results obtained by Hopkins and Moore (36). The technical implementation of HTA-CR neuromodulation caused no problem.

The primary objective of the study was to evaluate whether the adapted stimulation with HTA-CR neuromodulation leads to considerable tinnitus improvements. Our results show that despite the small number of patients (*n* = 25) and shorter duration of the study (i.e., ~4 months) HTA-CR neuromodulation has a remarkable response rate of 72%. A similar response rate was seen with standard CR neuromodulation only in the first trial (28) or after 6 months (31, Wurzer et al. submitted). Moreover, most of the patients reported first subjective changes, e.g., subjective change of tinnitus frequency, already after 2–3 weeks whereas such observations were reported not until 6–8 weeks with standard CR neuromodulation (31, Wurzer et al. submitted).

Unfortunately, the modified stimulation caused a temporary deterioration of tinnitus symptoms in two patients; another control examination after study end revealed that their TF scores were similar to before study start. Based on these first observations it seems reasonable to assume that HTA-CR neuromodulation might induce accelerated changes of tinnitus symptoms as compared to standard CR neuromodulation whereas the overall improvements are similar.

We also reported here a 10.9% improvement of TF score from screening visit 0 to visit 1. This change occurred before therapy start and reflects positive expectations on the therapy outcome. However, most studies do not report detailed data on this “pre-study effect” although it is well-known from clinical experience. Importantly, the effect of HTA-CR neuromodulation reported in our study was calculated as TF score difference between baseline visit 1 (mean TF score: 44.9 ± 12.1 points) and end of study visit 4 (mean TF score: 32.6 ± 15.5 points) resulting in a mean TF score change of 12.3 points. If we consider the TF scores from screening (mean TF score: 50.4 ± 12.9 points), the overall result would even be better resulting in a mean TF score change of 17.8 points and a response rate of 88% (i.e., 60% “Winners” plus 28% “Responders”). Only for 3 of 25 patients (12%) HTA-CR neuromodulation would result in no improvement of their tinnitus symptoms.

In summary, the results of this small feasibility study suggest that the new HTA-CR neuromodulation is at least comparable to the standard CR neuromodulation and might provide another therapeutic option in the treatment of chronic tonal tinnitus.

## Data availability

The datasets generated and/or analyzed during the current study are available from the corresponding author on reasonable request.

## Author contributions

HW planned the study, executed the study and wrote the manuscript together with the second author. CH designed the adapted therapy, planned the study, supported the execution of the study and wrote the manuscript together with the first author.

### Conflict of interest statement

HW is an ENT physician with a tinnitus clinic in Munich, Germany and also a consultant for Brook Henderson Group (Desyncra Technologies Ltd.) since 2014 (Medical Advisor Germany). CH is employed by Desyncra Operating GmbH, Bad Neuenahr-Ahrweiler (part of Desyncra Technologies Ltd, London, UK) and worked with Juelich Research Center between 2002 and 2016, and has received research funding from the European Community, the Federal Ministry of Education and Research (Germany), the Deutsche Forschungsgemeinschaft, and the Helmholtz Association.
